# Characterization of the MeCP2^R168X^ Knockin Mouse Model for Rett Syndrome

**DOI:** 10.1371/journal.pone.0115444

**Published:** 2014-12-26

**Authors:** Eike Wegener, Cornelia Brendel, Andre Fischer, Swen Hülsmann, Jutta Gärtner, Peter Huppke

**Affiliations:** 1 University Medical Center Göttingen, Department of Child and Adolescent Health – Division of Neuropediatrics, Göttingen, Germany; 2 German Center for Neurodegenerative Diseases, Göttingen, Germany; 3 University Medical Center Göttingen, Department for Psychiatry and Psychotherapy, Göttingen, Germany; 4 Center for Nanoscale Microscopy and Molecular Physiology of the Brain (CNMPB), Göttingen, Germany; National Institute of Health, United States of America

## Abstract

Rett syndrome, one of the most common causes of mental retardation in females, is caused by mutations in the X chromosomal gene *MECP2*. Mice deficient for MeCP2 recapitulate some of the symptoms seen in patients with Rett syndrome. It has been shown that reactivation of silent *MECP2* alleles can reverse some of the symptoms in these mice. We have generated a knockin mouse model for translational research that carries the most common nonsense mutation in Rett syndrome, R168X. In this article we describe the phenotype of this mouse model. In male MeCP2^R168X^ mice life span was reduced to 12–14 weeks and bodyweight was significantly lower than in wild type littermates. First symptoms including tremor, hind limb clasping and inactivity occurred at age 27 days. At age 6 weeks nest building, rotarod, open-field and elevated plus maze experiments showed impaired motor performance, reduced activity and decreased anxiety-like behavior. Plethysmography at the same time showed apneas and irregular breathing with reduced frequency. Female MeCP2^R168X^ mice showed no significant abnormalities except decreased performance on the rotarod at age 9 months. In conclusion we show that the male MeCP2^R168X^ mice have a phenotype similar to that seen in *MECP2* knockout mouse models and are therefore well suited for translational research. The female mice, however, have a much milder and less constant phenotype making such research with this mouse model more challenging.

## Introduction

Rett Syndrome (RTT, OMIM #312750) is a neurodevelopmental disorder which occurs almost exclusively in females affecting 1∶10.000–1∶15.000 live births [1], [2]. The disorder is caused by mutations in the *MECP2* gene coding for the methyl-CpG-binding protein 2 (MECP2, OMIM #300005) [3]. After a period of normal development lasting 6–18 months developmental stagnation occurs followed by regression that mainly affects hand function and speech. The condition then stabilizes for many years. Other typical clinical features include hand stereotypies, gait apraxia, breathing abnormalities, anxiety, seizures and scoliosis [4], [5], [6], [7]. Mouse models deficient for MeCP2 recapitulate clinical features observed in human patients including gait apraxia, breathing abnormalities and the delayed appearance of symptoms [8], [9]. Experiments in conditional mouse models have demonstrated that it is possible to reverse the symptoms at a later stage of the disease by re-expression of MeCP2 [10]. This finding has stimulated research aimed at developing a cure for Rett syndrome. Studies in mice have shown positive effects of a growing number of compounds including desipramine, Ampakine, IGF-1, NO-711, 7,8-DHF, LM22A-4, choline, corticosterone, acetyl-L-carnitine, CNF-1 and fingolimod (recently reviewed in [11]). We have shown recently that it is possible to induce readthrough of nonsense mutations in the *MECP2* gene in vitro using different aminoglycosides [12]. To be able to test this therapeutic approach in vivo we generated a knockin mouse carrying the R168X mutation in *Mecp2*, the most common nonsense mutation in humans [13]. Here we present the characterization of this mouse line.

## Results

### General appearance of MeCP2^R168X/y^ mice

**Figure 1:**
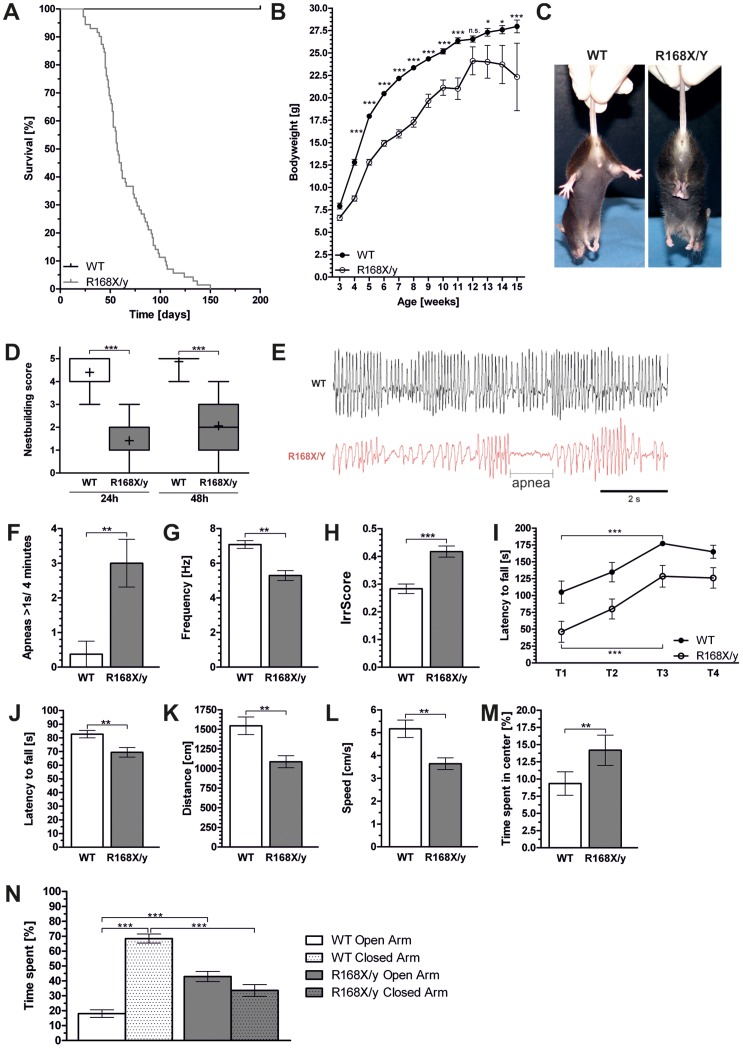
Characterization of MeCP2^R168X/y^ mice. Analysis of survival included 71 mutant mice (A). Body weight was measured weekly and compared to wild type littermates (B). Occurrence of hind limb clasping was measured weekly (C). Nest building was analyzed after 24 and 48 hours and scored according to Deacon 2006. Data were shown as box plots with median (−), mean (+) and whiskers indicating 5–95 percentile. (D). Plethysmography (E) was performed to analyze occurrence of apnea (F) as well as breathing pattern including the frequency (G) and the irregularity score (IrrScore) (H). Rotarod was used to analyze motor coordination. Latency to fall was measured on 2 consecutive days (T1–T4) at constant speed (I) and on 2 consecutive days with accelerating speed (J). To analyze locomotion open field test was performed showing total distance traveled and speed during a 5 minute period (K, L). To test anxiety time in the center of the open field (M) and time spend in the open arms of the elevated plus maze (N) was analyzed. Denotation of significance levels: * = p<0.05, ** = p<0.001 and *** = p<0.0001.

Appearance of mutant offspring was normal at birth and genotype and gender were distributed according to Mendel's laws. Median life span of MeCP2^R168X/y^ mice was significantly reduced to 57 days (p = 0.0311, n = 71, log-rank (Mantel-Cox test) with a range from 23 to 150 days (Fig. 1A). In the first two weeks mutant male mice showed normal growth compared to wild type littermates. Mutant male mice later exhibited significantly decreased body weight (p<0.001 for weeks 3–11 and week 15, p<0.01 for weeks 13 and 14, 2way ANOVA with Bonferroni post-test; n_R168X/y_ = 8 to 96, n_WT_ = 29 to82; Fig. 1B). At a mean age of 47 days (range: 27 to 78 days) 65% of mutant male mice (n = 96) showed hind limb clasping that persisted until death (Fig. 1C). Tremor was present in 63% with a mean start at age 48 days (range: 27 to 77 days).

### Impaired nest building in MeCP2^R168X/y^ mice

For small rodents nests are very important for breeding and protection against predators and other environmental factors, e.g. extreme temperatures. Therefore both male and female mice will build nests if nesting material is offered. Here we used autoclaved paper towels as nesting material. Male wild type mice (n = 15) immediately began to examine inserted nesting material. After a short exploration period wild type males started to build nests and almost finished them during the first hours. In contrast mutant males (n = 17) did not react to insertion of nesting material for at least 10 minutes and up to one hour after which they started to investigate the material. After 24 hours wild type males showed structured nests with a median score of 4 (min = 3, max = 5) while MeCP2^R168X/y^ mice showed a significantly lower median nest quality of 1 (min = 0, max = 3; p<0.0001). Even after 48 hours the majority of mutant mice failed to build complex structured nests with some mice leaving the material untouched. This resulted in a median nest quality of 2 (min = 0, max = 4) for MeCP2^R168X/y^ mice in contrast to wild type mice who had a median nest quality of 5 (min = 4, max = 5) at this point of time (Fig. 1D).

### Abnormal breathing in MeCP2^R168X/y^ mice

During home cage observation breathing abnormalities were observed in MeCP2^R168X/y^ mutant males. To analyze breathing, six week old male mice (n_WT_ = 8, n_R168X/y_ = 13) were analyzed by whole-body-plethysmography (Fig. 1E) revealing a highly increased occurrence of apneas in MeCP2 deficient male mice, which occur very rare in wild type mice (Fig. 1F, mean_R168X/y_ 3/4 minutes ±0.68 SEM, mean_WT_ 0.38/4 minutes ±0.38 SEM; p = 0.0046, Mann-Whitney test). Mutant males exhibited a significant decreased respiratory rate (Fig. 1G) of 5.29 Hz (±0.29 SEM) compared to 7.08 Hz (±0.23 SEM) in wild type animals (p = 0.0021, Mann-Whitney test) and a more irregular breathing pattern (Fig. 1H) represented by the IrrScore of 0.42 (±0.02 SEM) compared to healthy littermates with an IrrScore of 0.25 (±0.02 SEM; p = 0.0010, Mann-Whitney test).

### Abnormal motor function in MeCP2^R168X/y^ mice

To test the motor function rotarod and open-field test were performed with 23 wild type and 19 MeCP2^R168X/y^ male mice. During the first four days on the rotating rod mutant male mice showed a decreased performance compared to their healthy littermates. However, during the training period (training 1 vs. training 3) (Fig. 1I) the mutant mice showed an increase in rotarod performance (R168X/y_Training 1_ = 46.21 s±15.49 SEM and R168X/y_Training 3_ = 128.50 s±15.99, R168X/y_increase_ = 178%; p<0.001 repeated measure (mixed model) ANOVA) at constant speed like the wild type mice (WT_Training 1_ = 105.10 s±16.49 and WT_Training 3_ = 177.20 s±2.77, WT_increase_ 69%; p<0.001 repeated measure (mixed model) ANOVA). Nonetheless MeCP2 deficient males failed to reach wild type levels and showed no further increase during the last training session. When tested on an accelerated rotating rod mutant male mice showed a significantly shortened latency to fall compared to their healthy wild type littermates (latency to fall_R168X/y_ = 69.42 s±3.46 SEM and latency to fall_WT_ = 82.67 s±2.68, p = 0.0027) (Fig. 1J). In the open-field test MeCP2^R168X/y^ males showed a significant decrease in travelled distance (Fig. 1K, p = 0.0025, unpaired t test) of 1087 cm (±76.72) compared to 1544 cm (±112.20 SEM) and in average speed (Fig. 1L) (p = 0.0028, unpaired t test) 3.64 cm/s (±0.25 SEM) compared to 5.17 cm/s (±0.37 SEM) which is in agreement with the decreased motor coordination seen in the rotarod experiment.

### MeCP2^R168X/y^ mice show decreased anxiety related behavior

To determine anxiety related behavior the time spent in the center of the open-field was measured. Here the mutant males spent significantly more time in the center of the maze compared to healthy mice (time_R168X/y_ = 14.18%±2.19 SEM to time_WT_ = 9.33%±1.71 SEM, p = 0.0487, Mann-Whitney test; Fig. 1M). To confirm this finding we tested all mice in the elevated plus maze experiment as a standard task for anxiety like behavior. Here MeCP2^R168X/y^ males spent similar periods of time in open and closed arms (time_open arm_ = 42.90%±3.44 SEM and time_closed arm_ = 33.55%±3.95 SEM), whereas wild type animals show a preference for the closed arms (time_open arm_ = 18.08%±2.57 SEM to time_closed arm_ = 68.41%±3.03 SEM, p<0.0001, one-way ANOVA with Bonferroni's post test) (Fig. 1N). Consequently MeCP2^R168X/y^ mice spent significant more time in the open arms (time_R168X/y_ = 45.90%±3.44 SEM and time_WT_ = 18.08±2.57 SEM, p<0.0001, one-way ANOVA with Bonferroni's post test) and significant less time in the closed arms (time_R168X/y_ = 33.55%±3.95 SEM and time_WT_ = 68.41%±3.03 SEM) of the apparatus (Fig. 1N). Average speed and the number of visits in the open and closed arms were equal in both genotypes (not shown) indicating that the observed behavior is a consequence of an altered anxiety like behavior rather than impaired locomotion.

### General appearance of MeCP2^R168X/x^ mice

**Figure 2:**
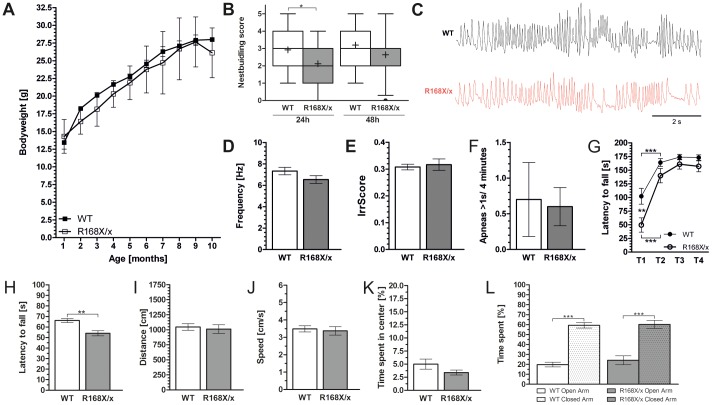
Characterization of the MeCP2^R168X/x^ mice. Development of bodyweight to age ten months (A). Nest building score after 24 and 48 hours. Data were shown as box plots with median (−), mean (+) and whiskers indicating 5–95 percentile (B). A representative plethysmography record, the breathing frequency and the irregularity score (IrrScore) (C–F). Rotarod at constant speed on 4 consecutive trials and on the accelerating rod (G, H). Results from the open-field test including distance traveled and average speed (I, J). Analysis of anxiety related behavior including time spend in the center at the open field and in the open arms in the elevated plus maze (K, L). Denotation of significance levels: * = p<0.05, ** = p<0.001 and *** = p<0.0001.

Similar to their male mutant littermates MeCP2 deficient females had a lower body weight than the wild type mice but this difference failed to reach statistical significance (Fig. 2A). Tremor and hind limb clasping was observed in 100% of all MeCP2^R168X/x^ mice (n = 31) starting at 36 to 86 days, with a mean of 55 days (tremors) and 53.5 days (hindlimb clasping). MeCP2 deficient females showed also gait ataxia, but less severe than their MeCP2^R168X/y^ littermates.

### Decreased nest building abilities in MeCP2^R168X/x^ mice

MeCP2 deficient females (n = 25) showed a significantly reduced median nest quality of 2 (min = 0, max = 4) after 24 h compared to the wild type mice (median = 3, min = 1, max = 5, n = 38, p = 0.0126, unpaired t test). After 48 h however, median nest quality was identical in both groups (median = 3) (Fig. 2B).

### No breathing abnormalities in MeCP2^R168X/x^ mice

Female MeCP2 deficient mice (n = 10) did not show abnormalities in whole body plethysmography, breathing rate (WT = 7.33 Hz±0.34 SEM, R168X/x = 6.54 Hz±0.36 SEM, p = 0.1287 unpaired t-test), irregularity (WT = 0.31±0.01, R168X/x = 0.32±0.02, p = 0.4359 Mann-Whitney test) or general occurrence of apneas (WT = 0.7 apneas/4 minutes±0.52, R168X/x = 0.6 apneas/4 minutes±0.27, p = 0.5435 Mann-Whitney test) (Fig. 2C–F) compared to wild type animals (n = 10). Impaired motor coordination in MeCP2R168X/x mice: As observed in mutant male mice heterozygous females (n = 23) showed a higher increase in performance (182,43%, p<0.001, one-way ANOVA with Bonferroni's post test) compared to their wild type littermates (60.47%, n = 36, p<0.001, one-way ANOVA with Bonferroni's post test) on the rotarod at constant speed, but in contrast female mice showed that increase already in the second session (training 1 vs. training 2) (Fig. 2G). On an accelerated rod heterozygous females showed a decreased performance compared to their wild type littermates (latency to fall_R168X/x_ = 53.99 s±2.41 SEM, n = 36 and latency to fall_WT_ = 66.15 s ±1.84 SEM, n = 23, p<0.0001, unpaired t test) (Fig. 2H). During the open-field test mutant females (n = 23) showed a normal traveled distance (Fig. 2I) accompanied by a normal mean speed (Fig. 2J) compared to their healthy littermates (n = 37).

### MeCP2^R168X/x^ mice show normal anxiety related behavior

During open-field test heterozygous females spend a comparable amount of time in the center of the maze as wild type mice (time_R168X/x_ = 3.37±0.47 and time_WT_ = 4.98 s±0.96, Fig. 2K). Consistently they showed a preference for the closed arms in the elevated plus maze (time_WT_ = 59.24 s±2.81 SEM and time_R168X/x_ = 60.20 s±4.14 SEM) (Fig. 2L).

## Discussion

In 2011 the National Institute of Neurological Disorders and Stroke (NINDS), the Eunice Kennedy Shriver National Institute of Child Health and Human Development (NICHD), the International Rett Syndrome Foundation (IRSF) and the Rett Syndrome Research Trust (RSRT) held a meeting to evaluate best practices for the use of animal models in preclinical evaluation of potential new RTT therapeutics. It was concluded that it is necessary to generate and characterize Mecp2 alleles that model the most common human RTT mutations and to develop a detailed characterization of female heterozygous mice carrying different Mecp2 alleles [14].

So far 12 different mouse models for Rett syndrome have been generated [14]. The majority do not carry Mecp2 alleles that model the most common human RTT mutations. We here present the results of the characterization of the MeCP2^R168X^ mouse model that we generated for translational studies on readthrough of nonsense mutations in the *MECP2* gene. In this mouse a R168X mutation was introduced, the second most common mutation found in patients with Rett syndrome and the most common nonsense mutation. We have previously reported that in this mouse model no full-length or shortened MeCP2 can be detected [13].

The parameters and experiments used in this study have been chosen because they are widely used and well standardized and they showed robust, reproducible results thereby facilitating the reproduction of translational experiments in independent laboratories.

In all mouse models that are expected to be associated with a complete loss of function of MeCP2 survival has been found to be reduced in male mice to less than 12–14 weeks [8], [9], [15], [16], [17], [18], [19] Consistent with these findings 90% of MeCP2^R168X/y^ mice died before age 14 weeks. While survival seems to be relatively independent of the genetic background the development of bodyweight is not [14]. Mice on a C57BL/6 background including MeCP2^R168X^ mice have a decreased bodyweight while the mice on a 129 background often show an increase. As described in other RTT mouse models MeCP2^R168X/y^ mice exhibited a normal early development followed by the onset of RTT like symptoms including hind limb clasping, spontaneous tremors and progressive inactivity after day 27 [8], [9], [18], [19], [20]. Consequently behavioral and physiological experiments were performed at 6 weeks of age.

Rotarod was used to analyze motor coordination that is always disturbed in Rett syndrome patients. MeCP2^R168X^ male and female mice showed impaired motor performance on the accelerating rotarod (Fig. 1J and Fig. 2H) which seems to be a robust finding in all mouse models without functional MeCP2 [14], [17], [18], [21], [22], [23]. Interestingly, we found during the 4 trial training period at constant speed a strong increase in rotarod performance in the mutant mice of both genders indicating some learning capacity. To analyze general locomotor activity the open-field test was used. As seen in other RTT mouse models, the MeCP2^R168X/y^ mice activity was significantly reduced with decreased average speed and distance traveled [8], [9], [16], [18], [21], [22].

In the open-field test it was also seen that the MeCP2^R168X/y^ mice spend significantly more time in the center of the open field indicating reduced anxiety-like behavior. To confirm this finding we used the elevated plus maze where the mice again spent significantly more time in the open arms. It was excluded that this was due to reduced locomotor activity by analyzing the total number of visits in the center and the average speed. While decreased anxiety-like behavior has also been a finding in other mouse models of RTT it is not typical for patients with RTT. [18], [19], [22], [24]. Contrary in patients with RTT increased anxiety has been described [25].

Breathing irregularities including apneas during the active phase are a common feature in RTT patients [6]. As described for other mouse models deficient for MeCP2, MeCP2^R168X/y^ mice showed reduced respiratory frequency, irregular breathing and apneas [14], [26], [27], [28], [29]. In the male mice breathing abnormalities seem to be a very robust finding independent of the genetic background.

Another test that produced very consistent abnormal findings was nest building. Rodents of both genders naturally build nests for breeding and protection, environmental changes and hypothermia [30], [31]. We found that MeCP2^R168X/y^ mice displayed a significantly reduced ability to build complete nests which is in agreement with results in other RTT mouse models [20], [32], [33]. However, one has to keep in mind that nest building is a very complex task that is not just influenced by motor coordination but also by motor activity and hormonal influences [31].

Although RTT almost exclusively occurs in females the vast majority of translational studies so far have been performed in male mice because they have an earlier, more severe and more constant phenotype. This was also the case in MeCP2^R168X^ mice. Body weight was not significantly reduced in the female mice while tremor and hind limb clasping was more penetrant in the females and occurred not much later than in the male mice. Nest building was delayed but reached the same median nest quality of healthy littermates after 48 h. Breathing that has been reported to be abnormal in other female Rett mice [34], [35], [36] was normal in MeCP2^R168X/x^ mice. Furthermore we did not find abnormalities in the anxiety-like behavior and general locomotion. However, we only performed a 5 minute open field test. As the novelty of the open field can induce motor activity we cannot exclude that a longer open field test might have shown abnormalities. The only consistent finding in the female mutant mice was impaired motor coordination on the rotarod.

Recently, the behavioral characterization of another mouse model carrying the same mutation has been described [37]. In this model a BspHI restriction site resulting in a second stop codon was introduced after amino acid 168 (MeCP2^R168XBspHI^) making it not suitable for studies on readthrough of nonsense mutations because the readthrough product would not be a functional MeCP2 protein. Furthermore this mouse model is maintained on a different genetic background (C57BL/6 X 129S6/SvEv) compared to the mouse described in this article. It has been described that expression of the same Mecp2 allele on different genetic backgrounds can confer significant differences in phenotypic effects [14]. However, both in mouse models the males have a very similar phenotype regarding life span, body weight, motor function and anxiety–like behavior [37]. The different genetic background might account for differences seen in the females. While the females of both genotypes show a decreased performance on the rotarod only the MeCP2^R168XBspHI^ showed a reduced body weight [37]. Furthermore the breathing phenotype differs between female MeCP2^R168XBspHI^ and MeCP2^R168X^ mice. While female MeCP2^R168XBspHI^ mice were described to show an increased incidence of apneas, irregular breathing and reduced respiratory frequency, we did not observe any abnormalities in the female MeCP2^R168X^ mice when compared to wild type littermates [38].

In conclusion male MeCP2^R168X^ mice recapitulate the phenotype seen in the majority of RTT mouse models. As this model carries the second most abundant mutation observed in Rett syndrome patients it is well suited for translational research especially on readthrough of nonsense mutations. Unfortunately female MeCP2^R168X^ mice do show a severe phenotype and research will therefore be performed primarily with male mice. One has to be aware that this is a serious limitation because *MECP2* is an X chromosomal disorder and the vast majority of RTT patients are females and therefore mosaic for the MeCP2 defect.

## Material and Methods

### Animals

Generation of the transgenic mice has been described elsewhere [13]. As animals that contained a FRT-flanked Neo-cassette showed very low breeding performance, they were crossed with 129S4/SvJaeSor-*Gt(ROSA)26Sor^tm1(FLP1)Dym^*/J mice to achieve a germ line removal of the Neo-cassette. Heterozygous mice missing the Neo-cassette were then backcrossed for ten generations to C57BL6/J. To obtain offspring for all experiments heterozygous *Mecp2^R168X/x^* females were mated with wild type C57BL6/J males to obtain *Mecp2^+/y^*, *Mecp2^R168X/y^*, *Mecp2^+/+^* and *Mecp2^R168X/x^* littermates. All animals were euthanized with carbon dioxide. Mice were housed on a 12:12 hour light:dark cycle without any environmental enrichment except autoclaved paper towels as nesting material. Food and water was supplied ad libitum. The study was approved by the Animal Care Committee of the University Medical Center Göttingen (UMG) and by the “Niedersächsische Landesamt für Verbaucherschutz und Lebensmittelsicherheit” (LAVES). All animals were examined daily by animal care takers. Mice which were hypoactive and lost more than 20% bodyweight in 48 hours where euthanized by use of carbone dioxide. For all behavioral tests mice in symptomatic stages (six weeks for males and nine months for females) were used. Behavioral tests were performed in the following order within a 24 h interval: elevated plus maze, open field, rotarod, nest building and plethysmography. During the behavioral experiments mice died or were excluded for the rest of the testing battery because of unexpected impairments, e.g. epileptic seizures during rotarod training. Therefore numbers of mice were decreasing through-out the experiments.

### Assessment of mice for general appearance

Mice were examined three times a week. During the examination process mice were lifted for the detection of hind limb clasping and weighed subsequently. Tremors were assessed tactually and visually.

### Survival analysis MeCP2^R168X^ mice

Animals used for survival studies were examined daily by animal care takers. To support strengthening of weak mice, daily changed paste, of normal food pellets, was provided in addition to pellets. No humane endpoints were used, but mice which were almost complete hypoactive and loose more than 20% bodyweight in 48 hours where euthanized by use of carbone dioxide. Neither analgesics nor anaesthetics where used due to absence of evidences of pain.

### Measurement of ventilation

Ventilation was measured by whole-body-plethysmography measuring pressure changes resulting from the warming of the inspired air and cooling during expiration [39]. WT and Mecp2^R168X/y^-Mice were placed in a plexiglas chamber (300 ml volume, custom made) that was connected to a differential low-pressure transducer (model DP1 03, Validyne Engineering, Northridge, CA). The second channel of the pressure transducer was connected to a reference chamber (300 ml). The signal from the pressure transducer was fed into a sine wave carrier demodulator (CD-15, Validyne Engineering). For the analysis, pressure fluctuations were Band-Pass filtered (1.5–500 Hz), amplified (four times) before storing on an Apple-PC computer. For digitization (1 kHz sampling rate) an ITC-16 interface (InstruTECH/HEKA, Lambrecht) was used which was controlled by Axograph 4.8 software (Axon Instruments, Foster City, CA). Since animals were allowed to explore the chamber freely, some pressure changes resulted from temperature changes during sniffing. To prevent accumulation of CO_2_ in the chamber, we introduced a bias flow using the 150 ml/min suction of a Normocap CO_2_-sensor (Datex, Instrumentarium Oy, Helsinki, Finland). CO_2_ concentration could be kept below 3%. For the older female mice a modified set up was used with a larger chamber and different bias flow (2 l/min). Pressure changes were detected with TRD5700 Pressure Transducer (Buxco) and stored on Windows-PC-computer running Ponemah software (DSI, St. Paul, MN, USA). Pressure measurements from both setups were exported and converted to axon binary files to use the same analysis protocols and software. Breaths from a period of 4 min after at least 10 min adaptation to chamber were analyzed automatically by the threshold search event detection method of Axon clampfit 10.3″ (Molecular Devices, Sunnyvale, CA). Breathing frequencies were calculated as the reciprocal of the averaged inspiratory interval. The number of inspiratory intervals that were longer than 1 s was determined during the 4 min as a parameter for central apneas (Stettner et. al., 2008) Additionally, an irregularity score (IrrScore) was determined (IS = 100*ABS (Int_n_- Int_n-1_)/Int_n-1_) for each respiratory cycle [40], [41].

### Open-field test

Mice were placed in the open field (50 x 50 cm) and monitored for 5 minutes using VideoMot 2 software-camera system (TSE Systems GmbH, Bad Homburg, Germany). To analyze motor performance total distance travelled and the average speed were measured. The center-edge-ratio was used to determine anxiety.

### Elevated Plus Maze

To test anxiety related behavior elevated plus maze was used. The maze consists of two open arms (each 30 cm x 5 cm), two closed arms (each 30 cm x 5 cm) and a center platform (5 cm x 5 cm). Animals were monitored for 5 minutes with VideoMot 2 (TSE Systems GmbH, Bad Homburg, Germany). To determine anxiety related behavior times spend in open and closed arms.

### Rotarod

Motor coordination and motor learning were tested by accelerating rotarod paradigm, using Rotarod Advanced (TSE Systems GmbH, Bad Homburg, Germany). The experiment was done over 4 days, two sessions each day with a six hour rest period. On training days (days one and two) mice ran on the rod with constant speed of 5 rounds per minute (rpm) over 180 seconds. On testing days (days three and four) mice were tested on an accelerated rod starting at 5 rpm and accelerated up to 40 rpm over 240 seconds. For statistical analysis Kruskal-Wallis and one-way ANOVA test were chosen because of unequal sized groups in this experiment.

### Nest building

To assess nest building abilities mice were housed individually for at least 2 days in new cages to habituate to the new environment. The experiment ran over three days. On the first day the nesting material (autoclaved paper towel) was removed. One hour prior to dark phase a new paper towel was placed inside the cages. After 24 hours and 48 hours the nests were viewed and scored. Scoring was done according to Deacon 2006 [31]. Nest quality was assessed with the following scale: 0 =  untouched nesting material, 1 =  touched but unformed material (the nest itself was beneath the towel), 2 =  material was slightly formed (e.g. flipped edges), 3 =  nests showed a visible structure, 4 =  strongly increased complexity was observed and walls were established, 5 =  nests were completely circled and at least partially covered.

### Statistical analysis and graph representation

All statistical analysis and graphs were generated using GraphPad Prism5 Software. Graphs were presented with mean with SEM if not noted otherwise.

## Supporting Information

S1 ARRIVE Checklist (PDF)
